# Use of vacuum assisted closure in instrumented spinal deformities for children with postoperative deep infections

**DOI:** 10.4103/0019-5413.62067

**Published:** 2010

**Authors:** Federico Canavese, Joseph I Krajbich

**Affiliations:** Department of Orthopedics, Shriners Hospital for Children, 3101 SW Sam Jackson Park Road, Oregon 97239, Portland, USA

**Keywords:** VAC therapy, deep wound infection, spinal deformity

## Abstract

**Background:**

Postoperative deep infections are relatively common in children with instrumented spinal deformities, whose healing potential is somewhat compromised. Children with underlying diagnosis of cerebral palsy, spina bifida and other chronic debilitating conditions are particularly susceptible. Vacuum-assisted closure (VAC) is a newer technique to promote healing of wounds resistant to treatment by established methods. This article aims to review the efficacy of the VAC system in the treatment of deep spinal infections following spinal instrumentation and fusion in children and adolescents.

**Materials and Methods:**

We reviewed 33 patients with deep postoperative surgical site infection treated with wound VAC technique. We reviewed clinical and laboratory data, including the ability to retain the spinal hardware, loss of correction and recurrent infections.

**Results:**

All patients successfully completed their wound VAC treatment regime. None had significant loss of correction and one had persistent infection requiring partial hardware removal. The laboratory indices normalized in all but three patients.

**Conclusions:**

Wound VAC technique is a useful tool in the armamentarium of the spinal surgeon dealing with patients susceptible to wound infections, especially those with neuromuscular diseases. It allows for retention of the instrumentation and maintenance of the spinal correction. It is reliable and easy to use.

## INTRODUCTION

Deep infection following instrumented fusion in the management of scoliosis is uncommon. However, when it does occur, it can result in significant morbidity, costs, and compromise of the desired correction. As surgical and perioperative techniques advance, more severely involved patients with complex neuromuscular deformities and significant comorbidities have been considered candidates for extensive spinal deformity surgery. Surgical site infection rates of up to 20% for this non idiopathic scoliosis population have been reported in the literature.^1^–^5^ The rate of infection is, higher in patients with neuromuscular scoliosis such as cerebral palsy, myelomeningocele than in those with idiopathic scoliosis.^6^ Various treatment protocols for debridement, soft tissue management and antibiotic therapy have been recommended with mixed results.

The use of wound VAC (Vacuum-Assisted Closure^®^ -KCI, Inc, San Antonio, TX) therapy has gained increasing popularity in the management of acute, subacute and chronic wounds. The controlled application of sub- atmospheric pressure has been reported to reduce edema, improve blood flow, aid in the formation of granulation tissue and in the debridement of necrotic tissue, remove infectious material and act as a sterile barrier.^7^–^10^ Moreover, because the wound is sealed, the risk of contamination is reduced. The number of case reports and retrospective reviews has been steadily growing, and the authors of these reports describe expanding uses for this therapy for wound in children and adults. Widespread use of the VAC for complex soft tissue injuries have generally demonstrated accelerated wound healing compared to traditional methods.^6^,^11^–^13^ The goal of the present article was to review the efficacy of the VAC system in the treatment of deep spinal infections following spinal instrumentation and fusion in children and adolescents.

## MATERIALS AND METHODS

After Institutional Review Board approval, a retrospective chart and radiograph review of all cases of deep spinal infection that occurred between March 1997 and December 2008 was performed. We reviewed 33 cases of postoperative spinal infections treated with the VAC. Thirty patients had spinal deformities treated by posterior instrumented arthrodesis (n=21), or by a combination of anterior release and posterior spinal fusion and instrumentation (n=9). The spinal instrumentation used varied according to underlying pathology [Table 1]. Standard segmental instrumentation using dual rods, hooks and pedicle screws was used for non neuromuscular curves and segmental instrumentation into the pelvis for most of the neuromuscular curves. Twelve patients had spinal deformities treated by standard segmental instrumentation, 12 by Luque rods, 5 by Unit rods and 1 by growing rods. The remaining three, had vertical expander prosthetic titanium rib (VEPTR) application with the aim to treat early onset spinal deformities [Table 1]. Current treatment of early onset deformities is carried out either with specific orthopedic aids or with vertebral arthrodesis surgery. Arthrodesis is not the ideal treatment for skeletally immature patients, but remains an option when other major spine deformities have been addressed. A recent strategy involves performing surgery on patients with early onset scoliosis, but requires the need to assess the spinal deformity constantly and at the same time support the expansion of the thoracic cage. For this purpose, expansible instrumentations such as VEPTR or growing rods are in use, which could permit and support spine and chest growth.^14^–^16^

**Table 1:** Clinical details of patients

Gender	Diagnosis	Scoliosis type	Anatomical site	Surgery	Type of instrumentation

M	DMD	Paralytic	T-L	PSF	UR (T2-pelvis)
M	CP, spastic quad.	Neuromuscular	T-L	AR, PSF	LR (T10-L3)
F	VCF syndrome	Neuromuscular	T-L	PSF	SI (T9-L3)
F	PW syndrome	Neuromuscular	Kyphosis (T)	AR, PSF	LR (T4-L3)
F	LG syndrome	Paralytic	T-L	PSF	LR (T1-pelvis)
F	Myelodysplasia	Neuromuscular	T-L	AR, PSF	UR (T2-pelvis)
F	CP, spastic quad.	Neuromuscular	T-L	PSF	UR (T3-pelvis)
F	CP, spastic quad.	Neuromuscular	T-L	PSF	UR (T1-pelvis)
F	Infantile scoliosis	Idiopathic	T-L	AR, PSF	SI (T4-L3)
M	Myelodysplasia	Neuromuscular	T	AR, PSF	SI (T6-T12)
F	Myelodysplasia	Neuromuscular	T-L	AR, PSF	SI (T8-L3)
F	SMA 3	Paralytic	T-L	AR, PSF	LR (T2-pelvis)
F	Idiopathic scoliosis	Idiopathic	T-L	PSF	SI (T3-L1)
M	SMA 2	Paralytic	T-L	PSF	LR (T2-pelvis)
M	PB syndrome	Non idiopathic/Congenital	L	PSF	Growing rods
F	Transverse myelitis	Neuromuscular	T-L	VEPTR	VEPTR
F	Myelodysplasia	Neuromuscular	Kyphosis (T)	Kyphectomy	LR (T2-pelvis)
M	Unknown neurom. disease	Neuromuscular	T-L	PSF	SI (T3-L4)
F	Myelodysplasia	Neuromuscular	Kyphosis (T)	Kyphectomy	LR (T2-pelvis)
M	PB syndrome	Non idiopathic/Congenital	Kyphoscoliosis (T)	PSF	Fusion with VCR after Halo
F	Down syndrome	Non idiopathic	T-L	PSF	SI (T7-L2)
M	DMD	Paralytic	T-L	PSF	LR (T2-pelvis)
F	Idiopathic scoliosis	Idiopathic	T-L	PSF	SI (T3-L3)
F	Myelodysplasia	Neuromuscular	T-L	AR, PSF	SI (T10-L3)
F	CP, spastic quad.	Neuromuscular	T-L	AR, PSF	LR (T2-pelvis)
M	CP, spastic quad.	Neuromuscular	T-L	PSF	LR (T2-pelvis)
M	SMA 2	Paralytic	T-L	VEPTR	VEPTR
F	CP, spastic quad.	Neuromuscular	T-L	PSF	UR (T3-pelvis)
M	CP, spastic quad.	Neuromuscular	T-L	PSF	LR (T2-pelvis)
F	Myelodysplasia	Neuromuscular	Kyphosis (L) + T	Kyphectomy	LR (T2-pelvis)
M	SMA 2	Paralytic	T-L	VEPTR	VEPTR
F	SMA 3	Paralytic	T-L	PSF	SI (T2-L5)
F	Idiopathic scoliosis	Idiopathic	T	PSF	SI (T2-T10)

DMD: Duchenne muscular dystrophy; CP: Cerebral palsy; PW: Prader Willi syndrome; LG: Lennox-Gastaud syndrome; VCF: Velo-Cardio-facial Syndrome; SMA; Spinal muscular atrophy; PB: Prune belly syndrome); Anatomical site (T-L: Thoracolumbar; T: Thoracic; L: Lumbar); Type of surgery performed (AR: Anterior release; PSF: Posterior spinal fusion; ASF: Anterior spinal Fusion); Type of instrumentation implanted (UR: Unit rod; LR: Luque rods or SI: Standard segmental instrumentation; VCR: Vertebral column resection)

Depth of infection decided the need for VAC technique. Patients with only superficial infections were not treated with VAC and, therefore, not considered. Other exclusions included patients with anterior wound complications, and those who developed pressure wounds when treated with VAC not related to the surgical site. The discretionary power lay with the surgeon, based upon the macroscopic appearance of the wound, and the underlying diagnosis.

Four patients developed a delayed surgical site infection and the remainder (29) acute deep infections (within four weeks from the index procedure). All patients were treated with the same procedure. Most (n=19) of the patients had cognitive and functional compromise and were overweight. Children with myelodysplasia and spastic quadriplegia, with paralytic bladder or poor bladder control, were characterized by either chronic urinary tract colonizations and/or diaper use for bowel and bladder incontinence. Those patients who did not have initial application were treated with appropriate surgical debridement and closure over deep drains.

All patients were evaluated for the length of follow-up, need for hardware removal, infection eradication by clinical, radiographic and laboratory indicators such as C-reactive protein (CRP) and white cell count (WCC), spinal pseudoarthrosis, need for additional surgery after the VAC application, and any loss of correction or hardware loosening. Intraoperative debridement involved thorough lavage and removal of all macroscopic contamination, devitalized tissue and loose bone graft. In most cases no attempt was made to remove all of the bone graft. Intraoperative culture specimens were obtained prior to application of VAC.

### VAC system application: Surgical technique

**Figure 1:**
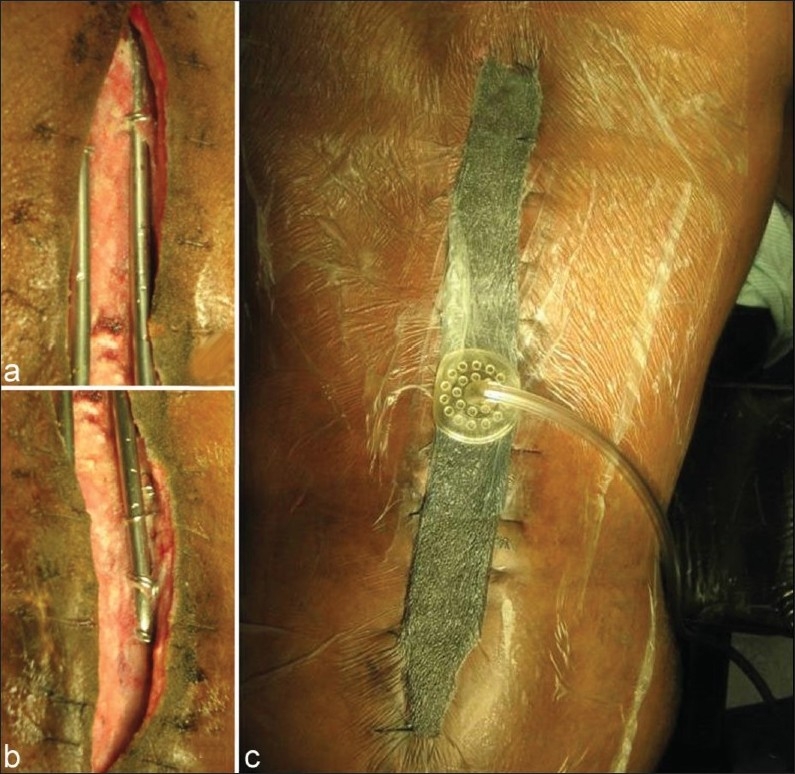
VAC system application. (a) and (b) show the upper and lower portion of the spinal wound. The hardware is in place. (c) shows the VAC system in place. The plastic sealant is applied several centimeters beyond the wound

The VAC system consists of an open-pored polyurethane ether foam sponge with 400 – 600 *µ*m pores, a connecting tube and a plastic sealant [Figure 1]. After thorough mechanical debridement of all nonviable tissue, the VAC sponge is cut and fitted into the wound. Metallic staples are used to secure the sponge to the skin and a plastic sealant is then used to cover the sponge. Attention is paid to apply the plastic sealant several centimeters beyond the margins of the wound to create an air-tight seal over the wound. A small hole (about 1 cm in diameter) is subsequently realized in the plastic sealant covering the sponge. A suction tube is then placed and fixed at this level and then connected to the negative pressure device. All patients in the present study had the sponge compressed under sub atmospheric pressure (125 mmHg), continuously or intermittently. The “controlled negative pressure” is used to evacuate wound edema, to increase blood flow, decrease bacterial load and increase the formation of granulation tissue.^7^–^10^ The VAC system is changed every three to four days.

## RESULTS

### Demographics and surgical procedures

There were 21 females (63.6%) and 12 males (36.4%) patients. The underlying diagnoses are outlined in Table 1. Twenty five patients were diagnosed with thoracolumbar (75.8%), two with thoracic (6.1%) and one with lumbar scoliosis (3%); three patients had thoracic kyphosis (9.1%), one thoracic kyphoscoliosis (3%) and one a combination of lumbar kyphosis and thoracic scolioisis (3%).

Posterior instrumented arthrodesis was performed in 18 patients (54.5%) and combined anterior release and posterior fusion and instrumentation in nine (27.3%). Three patients (9.1%) underwent kyphectomy and posterior spinal instrumentation and three patients had VEPTR implant (9.1%).

The average age at the time of chart review was 14.1±4.2 years (range 9 to 22). The average surgical time was 6.7 hours (range 3.5 to 11.5 hours). Drains (combination of superficial and deep) were used at the primary procedure in 20 of the 33 cases.

### VAC system application: Hardware retention

All patients presented with discharge from part of the wound; constitutional symptoms were present in 18 patients. The average time for presentation with postoperative acute deep spinal infections (n=29/87.9%) was 14±7.6 days (range 4-28 days). The four patients (12.1%) with postoperative chronic infection started symptoms on an average 7.6 months (range 1.5-12 months) after the primary surgery. No patients had exposed hardware at the time of initial presentation. All patients were managed with readmission, inflammatory marker evaluation, surgical debridement and application of VAC. All patients were started on dose-appropriate broad-spectrum antibiotics until sensitivities were available, at which time they were switched to a more specific antibiotic.

The VAC technique was applied at initial surgical debridement in majority of the patients. Two patients underwent one debridement prior to application, and two patients had application at the third procedure. These patients were treated at the beginning of our experience and presently we favor VAC application at initial debridement. The average number of surgeries required for change of VAC was 4.5±4.7 (range 1-20). The mean length of VAC application was 18.3±13.2 days (range 3-45) and the VAC dressing was changed on an average twice weekly. The average follow up was 3.7±2.7 years (range 1-9).

All patients received antibiotics for a period of at least six weeks based on the results of the wound culture sensitivities. The patients were maintained on antibiotics for the length of time the wound was open. Intravenous antibiotics were given for six weeks on average (range 1-12 weeks), and were followed by oral antibiotics for six months on average (range 4-12 months). The organisms included Staphylococcus *Areus* (n=9), *Escherichia coli* (n=5), *Pseudomonas Aruginosa* (n=4), *Enterococcus* Species (n=3), *Enterobacter* Species (n=3), *MRSA* (n=3), *Proteus mirabilis* (n=2), *Streptococcus pneumoniae* (n=1), *Streptococcus Agalactiae Group B* (n=1), *Candida Albicans* (n=1), *Klebsiella Pneumoniae* (n=1), *Propionibacteriae* (n=1), *Diphterioids* (n=1), *Bacteriodes Fragllis Group* (n=1), Prevotella Species (n=1) and no formal growth in eight patients. Nine patients (27.3%) had cultures positives for two or more pathogens.

Five patients were discharged with continued application as outpatients with VAC dressing changes as an outpatient procedure. The remaining patients were treated as inpatients because of logistics of long distance travel to our institution twice weekly, making more convenient for patients, and their families, to remain in the institution. Barring travel requirements most of these patients could have been treated as outpatient after first two or three wound VAC changes. The wounds were regularly reviewed for decrease slough and appearance of granulation tissue and eventually healing via secondary intention. No complications occurred related to usage of this system.

Although technical problems such as blockage of tubes, leaks or breakage of seals or ingrowth of granulation tissue into the polyurethane foam are possible; none of our patients had these complications as they were admitted in the hospital during this period. VAC dressing were changed every three to four days and all patients in the present study had the sponge compressed under sub atmospheric pressure (125 mmHg), continuously or intermittently.

Five patients required plastic surgery for definitive closure once the wound was clear of infection. We chose it to speed up the closure process to provide cover for the healthy granulation tissue rather than wait for natural epithelization while in the other cases we let the wound to granulate completely.

Most importantly, only one patient with acute surgical site infection required hardware removal for persistent infection. Two other patients had the hardware revised during follow up for hardware prominence and one of them required partial hardware removal distally. Consequently, there was no loss of correction of the spinal deformity [Table 2]. 

**Table 2:** Time of wound complication, number of VAC changes and need for hardware removal

Gender	Diagnosis	Time of wound complication**(days)**	Number of VAC changes	Hardware removal

M	DMD	9	2	No
M	CP, spastic quad.	26	3	Hardware revised then removed(1year after). No loss of correction
F	VCF syndrome	21	3	No
F	PW syndrome	10	2	No
F	LG syndrome	28	2	No
F	Myelodysplasia	23	3	No
F	CP, spastic quad.	7	1	No
F	CP, spastic quad.	11	10	No
F	Infantile scoliosis	1.5 mo	5	No
M	Myelodysplasia	15	3	No
F	Myelodysplasia	11	1	No
F	SMA 3	7	5	No
F	Idiopathic scoliosis	9	6	No
M	SMA 2	17	3	No
M	PB syndrome	9	4	No
F	Transverse myelitis	4	1	No
F	Myelodysplasia	5 mo	2	No
M	Unknown neurom. disease	10	1	No
F	Myelodysplasia	15	20	Yes. Partial hardware removal(distal). No loss of correction
M	PB syndrome	9	20	No
F	Down syndrome	12 mo	1	No
M	DMD	6	1	No
F	Idiopathic	28	1	No
F	Myelodysplasia	27	4	No
F	CP, spastic quad.	12 mo	5	Hardware revised (prominent) but not removed. No loss of correction
M	CP, spastic quad.	28	5	No
M	SMA 2	10	1	No
F	CP, spastic quad.	12	3	No
M	CP, spastic quad.	15	10	No
F	Myelodysplasia	7	8	No
M	SMA 2	5	2	No
F	SMA 3	14	3	No
F	Idiopathic scoliosis	14	6	No

mo: months

The average CRP and white cell count (WCC) at time of infection presentation was 6.3±5.5 mg/L and 11.1±3.3×10^3^ mL respectively; the average CRP and WCC after VAC applications and antibiotics treatment dropped to 1.1±1.4 mg/L and 7.2±3.2×10^3^ mL respectively. In three patients, with chronic urinary tract issues unrelated to spinal surgery, CRP remained elevated after the treatment: 2.3, 5.1 and 5.9 mg/L respectively.

## DISCUSSION

According to our experience, vacuum assisted closure therapy is a useful tool in the complex management of deep infections in pediatric spinal deformities as it improves the retention rate of hardware, which allow for maintenance of spinal correction and successful fusion.

VAC is a newer technique to promote healing for wounds resistant to treatment by established methods. Since introduction in 1997, the VAC has been used widely in the adult population.^11^–^13^ Its popularity has been due to the successful outcome reported in decreasing wound complications, lesser healing time and reduced overall morbidity to the patient. The first study reporting on the use of VAC therapy in children was published by Mooney *et al*. in 2000^17^ and since then data related to its usage in the pediatric population have been steadily growing.^11^,^17^–^21^

Mooney *et al*.^17^ reported successful results with the use of VAC technique in the pediatric population with extensive and complex soft tissue wounds. They demonstrated fewer dressing changes and potentially less extensive coverage procedures. With advances in anesthesia, intensive care and medical therapy, patients with more complex co morbidities are increasingly being considered for more extensive spinal intervention. One of the aims of using this technique in the pediatric patient is to potentially reduce the need for further complex soft tissue procedures, hardware removal with consequent loss of deformity correction and pseudoarthrosis. The use of the VAC is particularly appealing in the population of patients with multiple comorbidities, as it may obviate the need for complex flap surgery and later re operations. Patients requiring later re operations did not have the VAC system applied at initial debridement. However, these patients were treated at the beginning of our experience. Presently, we favor VAC application at initial debridement to avoid later surgeries.

Mehbod *et al*.^18^ reported successful results with use of VAC for the management of deep spinal infections in the adult population. They report clean closed wounds without hardware removal in twenty patients following deep spinal wound infection. In their series, approximately 2.2 (range 2 to 3) procedures were required until definitive closure, which occurred on average 7 days (range 5 to 14) after initial placement. Our patients had more levels fused than those reported by Mehbod *et al*. (13 vs 4) and most of our patients had application of the system after the initial debridement. Patients typically require dressing changes every three to four days. Mooney *et al*.^17^ reported that very young patients required VAC change more frequently because they experience a higher rate of tissue granulation formation. This high rate of granulation tissue formation is a potential complication as it can cause ingrowth into the pores of the polyurethane foam. There is no consensus on the amount of negative pressure that should be applied to wounds. Continuous or intermittent negative pressure of 75 mmHg is generally used in younger patients and 125 mmHg in older patients. All patients in the present study had the sponge compressed under sub atmospheric pressure (-125 mmHg), continuously or intermittently.

Picada *et al*.^19^ used a protocol of aggressive debridement and delayed primary or secondary closure to achieve clearance of infection in their series in 24/26 adult patients with deep infection after lumbosacral fusion. They had to remove the hardware in seven patients, in two of whom this was related to infection and in five to ‘localized discomfort’. Our study has demonstrated both a higher average number of procedures required and longer time for definitive wound closure compared to Mehbod *et al*.^18^ and Mooney *et al*.^17^ series. However, markers of inflammation and infection decreased after treatment in 90.9% of patients.

We attribute this to the intrinsic comorbidities (e.g. neuromuscular disorders, syndromic disorders, etc.) in our pediatric series. Most of our patients were non-ambulatory with limited ability to self-care. Furthermore, because of the nature of the spinal deformity, with instrumentation generally from the upper thoracic spine to the pelvis, our patients’ required longer incisions and a higher number of levels fused, compared with adult patients. This naturally predisposes to higher wound complication rates.^2^–^4^,^18^,^20^–^22^

Persisting deep infection often necessitates the removal of hardware.^21^,^22^ This presents a major problem in the scoliosis patient, with the potential for loss of correction.

Studies in the adult population^23^ have shown up 35.3% rates of hardware removal in cases of deep spinal infection. Although more recent studies have shown significantly lower rates necessitating hardware removal in adults, studies of early postoperative deep spinal infections always carry the risk of potential loss of correction due to hardware removal.^24^ In our series, only one patient (3%) required hardware removal for persistent infection and none of our patients had loss of correction as the hardware was left in place.

Richards *et al*.^21^ reported removal of hardware in all 10 patients in their series of deep infections following corrective scoliosis surgery. However, the average time to presentation following was 25 months (range 11 to 45) post-operatively. All but two cases achieved successful arthrodesis. Similarly, Soultanis *et al*.^25^ reported five cases of late deep infection in scoliosis patients. At the time of debridement, all patients had achieved successful arthrodesis, therefore the instrumentation was removed without complication. In their review of 26 cases of delayed surgical site infections after spinal deformity surgery, Hedequist *et al*.^5^ found that delayed surgical site infections need to be treated with implant removal to clear the infection. Patients may require repeat instrumentation and fusion at a later date if progressive deformity or symptomatic pseudoarthrosis developed after implant removal. Twelve of the 26 patients (46.2%) with delayed infections were treated with VAC but could not have the hardware retained. The authors concluded that in chronic infections the colonization of implants by bacteria cannot be eradicated without implant removal.^5^

Our experience is limited to early postoperative infection in which implant removal is undesirable as fusion has not been achieved. In conclusion, the VAC technique is a useful tool in the armamentarium of the spinal surgeon dealing with patients susceptible to wound infections, especially those with neuromuscular diseases. It allows for retention of the instrumentation and maintenance of the spinal correction. It is reliable and easy to use.
